# Comparison of standard and double reading and computer-aided detection (CAD) of interval cancers at prior negative screening mammograms: blind review

**DOI:** 10.1038/sj.bjc.6601356

**Published:** 2003-10-28

**Authors:** S Ciatto, M Rosselli Del Turco, P Burke, C Visioli, E Paci, M Zappa

**Affiliations:** 1 Centro per lo Studio e la Prevenzione Oncologica, Viale A. Volta 171, I-50131 Firenze, Italy; 2 Department of Radiology, Local Health Unit No.1, Turin, Italy

**Keywords:** breast cancer, diagnosis, mammography, screening, computer-aided detection

## Abstract

The study evaluates the role of computer-aided detection (CAD) in improving the detection of interval cancers as compared to conventional single (CONV) or double reading (DOUBLE). With this purpose, a set of 89 negative cases was seeded with 31 mammograms reported as negative and developing interval cancer in the following 2-year interval (false negative (FN)=11, minimal signs (MS)=20). A total of radiologists read the set with CONV and then with CAD. Overall, there were 589 cancer and 1691 noncancer readings with both CONV and CAD. Double reading was simulated by combining conventional readings in all 171 possible combinations of 19 radiologists, resulting in a total of 5301 cancer and 15 219 noncancer readings. Conventional single, DOUBLE and CAD readings were compared in terms of sensitivity and recall rate. Considering all 19 readings, cancer was identified in 190 or 248 of 589 readings (32.2 *vs* 42.1%, *χ*^2^=11.80, df=1, *P*<0.01) and recalls were 287 or 405 of 1691 readings (16.9 *vs* 23.9%, *χ*^2^=24.87, df=1, *P*<0.01) at CONV or CAD, respectively. When considering FN and MS cases separately, sensitivity at CONV or CAD was 50.2 or 62.6% (*χ*^2^=6.98, df=1, *P*=0.01) for FN and 22.3 or 30.7% (*χ*^2^=6.47, df=1, *P*=0.01) for MS cases, respectively. Computer-aided detection (average of 19 readings) was slightly and not significantly less sensitive (sensitivity: 42.1 *vs* 46.1%, *χ*^2^=3.24, df=1, *P*=0.07) but more specific (recall rate 23.9 *vs* 26.1%, *χ*^2^=3.8, df=1, *P*=0.04) as compared to DOUBLE (average of 171 readings). Average sensitivity for FN cases only was 62.6% for CAD and 64.8% for DOUBLE (*χ*^2^=0.32, df=1, *P*=0.58). Corresponding values for MS cases were 30.7% for CAD and 35.7% for DOUBLE (*χ*^2^=3.53, df=1, *P*=0.06). Compared to CONV, CAD allowed for improved sensitivity, though with reduced specificity, both effects being statistically significant. Computer-aided detection was almost as sensitive as DOUBLE but significantly more specific. Computer-aided detection might be used in the current practice to improve sensitivity of conventional single reading. Based on estimates of screening sensitivity and FN/MS interval cancer expected frequency, the absolute increase of screening sensitivity expected by introducing CAD-assisted reading may be estimated around 0.9%. The use of CAD as a possible surrogate to conventional DOUBLE needs to be confirmed by further studies, which should include a cost-effective analysis.

Screening mammography has been shown to be effective in reducing breast cancer mortality, although it is not very sensitive. Sensitivity estimates based on proficiency tests of screening mammography (Ciatto *et al*, 1996; Ciatto *et al*, 1999), or according to interval cancer proportional incidence, suggest that approximately one in four cancers may be missed by biennial mammographic screening in women aged 50–69 years (Paci *et al*, 1999). Adequate training is currently recommended to improve diagnostic accuracy, as well as double reading (DOUBLE) (Bird, 1990; Kirkpatrick *et al*, 1992; Thurfjell *et al*, 1994; Ciatto *et al*, 1995a, 1995b; Kopans, 1998). False negatives (FNs) may be caused either by misperception or by wrong analysis of perceived abnormalities (Bird *et al*, 1992; Dijk *et al*, 1993; Harvey *et al*, 1993; Kopans, 1998). Perception might be improved by computer analysis of digitalised images identifying mammographic sites for second review. Population-based screening implies a huge number of mammograms being read as well as a low prevalence of cancer, both conditions favouring readers' loss of attention and fatigue: this might represent an ideal setting for computer-aided detection (CAD).

The influence of CAD on diagnostic accuracy must be properly tested and demonstrated, before it may be introduced in the current practice. A blind study comparing conventional and CAD readings by the same operators is a simple method for short-term evaluation of CAD diagnostic impact. In a previous study (Ciatto *et al*, 2003), comparing conventional and CAD readings of a set of mammograms seeded with screening detected cancers, we observed that (a) CAD achieves a slight increase of sensitivity while slightly reducing specificity and (b) simulated conventional DOUBLE and single CAD readings results are comparable. In the present study, with a similar design, we tested the impact of CAD in a set of negative mammograms seeded with prior negative screening mammograms of interval cancers.

## MATERIAL AND METHODS

The study set consisted of 120 original mammograms drawn from Florence city screening programme archives. All subjects had been originally reported as negative. In total, 89 subjects were confirmed to be negative after 2 years, whereas 31 subjects developed a cancer (single lesion) in the following 2-year interval. Prior screening mammograms of interval cancers were selected from the Florence city screening programme interval cancer archive among consecutive cases classified as ‘false negative’ (FN) or ‘minimal signs’ (MS) according to the European Community guidelines (European Commission, 2001). Control negative cases were selected randomly from screening archives. The screening protocol in the Florence programme uses single oblique view mammography at repeat screening in nondense breasts. Single oblique view had been performed in the majority of interval cancers (18 of 31) and negative cases (63 of 89) selected for the study.

The CAD system tested was developed by CADx Systems Inc. (Beavercreek, OH, USA). Original films were digitalised at the Department of Radiology of the Turin Local Health Unit No. 1 in Turin by means of a CCD digitiser operating at 43.5 *μ*m per pixel. Digitised images were then submitted to computer analysis using iterative applications of embedded intelligent systems to identify mammographic locations warranting second review. Paper-printed images were produced with the indication of the site for second review (calcifications and opacities were identified on the print with different marks) selected by the computer.

Of the 32 radiologists currently involved in a population-based mammography screening programme in the Tuscany Region, 19 accepted the invitation to undergo the test and read the set, displayed on a rotating viewer. First, conventional reading (CONV) was performed, whereas a second reading session (CAD) was repeated with the help of CAD printouts. The second reading was performed 1–2 weeks after the first, blind of the first reading results. All radiologists were not informed of the results of both readings until the study was completed. Readers were invited to indicate abnormalities (breast and site) that they would have selected for diagnostic assessment by marking the lesion site on a prepared breast scheme. There was no special training in reading with the assistance of CAD printouts, apart from a short demonstration (immediately prior to CAD reading) by one of us (SC) who has a large experience with CAD-assisted reading. Test results were evaluated in terms of sensitivity and recall rate (determined only on noncancer cases =1−specificity). Sensitivity was determined on all cancers as well as on FN and MS cases, separately. Statistical analysis of differences in test results was based on *χ*^2^-test (cutoff for *P*-value=0.05). Overall, average results included 589 cancer and 1691 noncancer readings for each modality. Computer-aided detection was assumed to modify CONV report only in the sense of additional recall. Thus, CONV and CAD- results were compared considering that only cases reported as negative at CONV with CAD-marked lesions reported as positive at CAD might modify CONV report, whereas CONV-positive reports were automatically assumed as positive also at CAD. This is consistent with the intended use of CAD for minimising observation oversights by identifying areas on the mammogram for a second review. Computer-aided detection is not intended to alter the radiologist's recall decision if the system does not mark an area that the radiologist detected on initial mammography evaluation.

Independent DOUBLE was simulated by combining couples of CONV readings. This gave a final comparison of 19 CAD and 171 simulated DOUBLE. Double reading was simulated according to an ‘or’ fashion in which a case is positive if either one or the other reader calls it positive. Although there are other ways to arbitrate discordant DOUBLE (consensus, arbitration), which may be associated to different diagnostic accuracy, the ‘independent’ modality was adopted in order to maximise sensitivity and since it is the modality currently used in the Florence screening programme.

## RESULTS

The mammography series in this study consisted of 120 cases, including 31 interval cancers. Computer-aided detection marked 340 sites for second review (average 2.8 per case or 1.06 per film, 132 microcalcifications, 208 opacities). Computer-aided detection marked the cancer site at least in one view in 16 of 31 cases with a case-based sensitivity of 51.6%. At review, 43 abnormalities (41 opacities and two clusters of calcifications) were evident at the cancer site. On a film and lesion basis, CAD marked 16 of 41 (39.0%) opacities and two of two calcifications. On a case basis, CAD marked 10 of 11 FN (90.9%), five of 20 MS (25.0%) cancers cases, and 73 of 89 (82.0%) negatives.

Table 1

**Table 1:** Comparison of conventional (CONV) and computer-aided detection (CAD)-assisted reading diagnostic accuracy (589 cancer, 1691 noncancer readings)

	**Sensitivity % (31 cancer cases)**		**Sensitivity FN (11 cases)**		**Sensitivity MS (20 cases)**		**Recall rate % (89 negative cases)**	
**Reader**	**CONV**	**CAD**	**CONV**	**CAD**	**CONV**	**CAD**	**CONV**	**CAD**
1	32.2 (10)	38.7 (12)	45.4 (5)	54.5 (6)	25.0 (5)	30.0 (6)	13.4 (12)	15.7 (14)
2	22.5 (7)	32.2 (10)	36.3 (4)	63.6 (7)	15.0 (3)	15.0 (3)	13.4 (12)	20.2 (18)
3	29.0 (9)	29.0 (9)	54.5 (6)	54.5 (6)	15.0 (3)	15.0 (3)	19.1 (17)	21.3 (19)
4	29.0 (9)	32.2 (10)	54.5 (6)	54.5 (6)	15.0 (3)	20.0 (4)	16.8 (15)	19.1 (17)
5	41.9 (13)	48.3 (15)	63.6 (7)	63.6 (7)	30.0 (6)	40.0 (8)	10.1 (9)	12.3 (11)
6	45.1 (14)	45.1 (14)	54.5 (6)	54.5 (6)	40.0 (8)	40.0 (8)	20.2 (18)	21.3 (19)
7	41.9 (13)	58.0 (18)	72.7 (8)	90.9 (10)	25.0 (5)	40.0 (8)	15.7 (14)	29.2 (26)
8	16.1 (5)	22.5 (7)	45.4 (5)	54.5 (6)	0 (0)	5.0 (1)	8.9 (8)	10.1 (9)
9	29.0 (9)	32.2 (10)	54.5 (6)	54.5 (6)	15.0 (3)	20.0 (4)	15.7 (14)	22.4 (20)
10	25.8 (8)	38.7 (12)	45.4 (5)	63.6 (7)	15.0 (3)	25.0 (5)	15.7 (14)	23.5 (21)
11	29.0 (9)	54.8 (17)	36.3 (4)	63.6 (7)	25.0 (5)	50.0 (10)	22.4 (20)	31.4 (28)
12	35.4 (11)	64.5 (20)	36.3 (4)	72.7 (8)	35.0 (7)	60.0 (12)	22.4 (20)	35.9 (32)
13	22.5 (7)	25.8 (8)	36.3 (4)	45.4 (5)	15.0 (3)	15.0 (3)	13.4 (12)	16.8 (15)
14	32.2 (10)	51.6 (16)	36.3 (4)	63.6 (7)	30.0 (6)	45.0 (9)	15.7 (14)	25.8 (23)
15	41.9 (13)	54.8 (17)	54.5 (6)	81.8 (9)	35.0 (7)	40.0 (8)	23.5 (21)	29.2 (26)
16	35.4 (11)	45.1 (14)	54.4 (6)	63.6 (7)	25.0 (5)	35.0 (7)	21.3 (19)	32.5 (29)
17	29.0 (9)	32.2 (10)	63.6 (7)	72.7 (8)	10.0 (2)	10.0 (2)	10.1 (9)	16.8 (15)
18	48.3 (15)	54.8 (17)	72.7 (8)	72.7 (8)	35.0 (7)	45.0 (9)	23.5 (21)	31.4 (28)
19	25.8 (8)	38.7 (12)	36.3 (4)	45.4 (5)	20.0 (4)	35.0 (7)	20.2 (18)	39.3 (35)
								
Total	32.2 (190)	42.1 (248)	50.2 (105)	62.6 (131)	22.3 (85)	30.7 (117)	16.9 (287)	23.9 (405)

reports the results of CONV and CAD readings by 19 radiologists. Of the 19 radiologists, 17 had an increase of sensitivity at CAD reading, the relative increase ranging from 11 to 88%. All 19 radiologists had an increase in recall rate at CAD reading, the relative increase ranging from 5 to 94%. Considering all 19 radiologists, cancer was identified in 190 or 248 of 589 readings (32.2 *vs* 42.1%, *χ*^2^=11.80, df=1, *P*<0.01) and recalls were 287 or 405 of 1691 readings (16.9 *vs* 23.9%, *χ*^2^=24.87, df=1, *P*<0.01) at CONV or CAD reading, respectively. When considering FN and MS cases separately, sensitivity at CONV or CAD reading was 50.2 or 62.6% (*χ*^2^=6.98, df=1, *P*=0.01) for FN and 22.3 or 30.7% (*χ*^2^=6.47, df=1, *P*=0.01) for MS cases, respectively.

Table 2

**Table 2:** Comparison of conventional (CONV) and computer-aided detection (CAD)-assisted readings (589 cancer, 1691 noncancer readings) with simulated independent double reading (DOUBLE) (5301 cancer, 15 219 noncancer readings) with respect to diagnostic accuracy (all are positive readings)

	**Sensitivity %**	**Sensitivity FN %**	**Sensitivity MS %**	**Recall rate %**
CONV	32.2 (190)	50.2 (105)	22.3 (85)	16.9 (287)
CAD	42.1 (248)	62.6 (131)	30.7 (117)	23.9 (405)
DOUBLE	46.1 (2444)	64.8 (1220)	35.7 (1224)	26.1 (3987)

shows the results of comparing CAD to DOUBLE readings. Computer-aided detection (average of 19 radiologists) was slightly and not significantly less sensitive (sensitivity: 42.1 *vs* 46.1%, *χ*^2^=3.24, df=1, *P*=0.07) but more specific (recall rate 23.9 *vs* 26.1%, *χ*^2^=3.8, df=1, *P*=0.04) as compared to DOUBLE (average of 171 readings). Average sensitivity for FN cases only was 62.6% for CAD and 64.8% for DOUBLE (*χ*^2^=0.32, df=1, *P*=0.58). Corresponding values for MS cases were 30.7% for CAD and 35.7% for DOUBLE (*χ*^2^=3.53, df=1, *P*=0.06). CONV was significantly less sensitive (sensitivity 32.2 *vs* 46.1%, *χ*^2^=40.5, df=1, *P*=10^−6^) and more specific (recall rate 16.9 *vs*. 26.1, *χ*^2^=68.0, df=1, *P*=10^−6^) as compared to DOUBLE.

## DISCUSSION

The present study allows the evaluation of the performance of CAD in the reading of screening mammograms reading. CONV and CAD were compared in a test scenario, a condition (‘halo effect’) that implies major differences as compared to the current screening setting, such as a higher prevalence of seeded positive cases and increased reader's alertness (awareness of being tested usually biases towards higher sensitivity and lower specificity). This condition most likely biased the study results and thus is not necessarily predictive of what would happen in the current practice but may be valid to compare two different reading modalities, which were both exposed to the same bias. However, as this test scenario is expected to maximise CONV sensitivity, FNs after the radiologist's initial reading without CAD are fewer and the sensitivity improvement due to CAD might be underestimated. Nevertheless, such a condition makes the finding of an increased sensitivity with the use of CAD even more meaningful.

Another possible bias might be suggested as CAD reading always followed CONV reading. It might be suggested that whenever images are seen twice, it is possible that performance is better the second time simply due to the fact that images can be recalled. We do not expect it to be a major bias of the study: the reading of the set was rather fast, as in current screening practice, and even if some images could be recalled, recalling does not imply a change in accuracy, and it is unclear in which direction such a bias, if any, could go. However, a counterbalanced study design with CAD and CONV reading changing in order according to a random fashion was not possible, as the study was designed to evaluate the influence of CAD over CONV reading, and thus, CAD reading could not be performed at the first session.

The study design did not foresee any special training of radiologists in reading with the assistance of CAD produced printouts. It is likely that this modality of CAD-assisted reading would increase reading time with respect to conventional reading, and also that this effect may decrease with experience. This study did not take into account the effect of CAD-assisted reading on reading time, considering that the ideal setting for CAD use is digital mammography: with immediate and automatic display of CAD marks directly on the mammographic image on the monitor, the impact of CAD on reading time would be considerable reduced as compared to the indirect methodology used in the present study, using paper printouts. We have no data to prove or disprove that prior training in CAD-assisted reading may be associated to a different impact on sensitivity. In the present study, only two readers (nr. 18 and nr. 19) had been previously involved in CAD-assisted reading studies and thus had some experience as compared to other readers, but their performance in terms of absolute increase in sensitivity (nr. 18=+6.5%, nr. 19=+12.9%) was not significantly different from the average (*χ*^2^=0.002, df=1, *P*=0.97).

The modality of this study was relatively complex (digitalisation of conventional films and reading with the help of CAD printed images) and, although the system may be implemented in current practice at a sensible cost, the use of CAD seems ideal for digital mammography soft copy reading, with automatic display of CAD marks on the monitor. For this reason, no cost analysis was performed in the present study.

Interval cancers in the study set were different as to their ‘visibility’ on the prior screening mammogram. Cases classified as FN were those in which a mammographic abnormality warranting further assessment was clearly visible even at blind review (without the help of the diagnostic mammogram), whereas cases classified as MS showed possible subtle abnormalities (minimal) at the cancer site, better appreciable at informed review (with the help of the diagnostic mammogram). These two categories are currently used in the analysis of interval cancers and imply a different chance of detection, which is relevant for FN cases, most likely missed due to fatigue and loss of attention, and virtually null for MS cases, which are easily identified only in retrospect at ‘informed’ review. Attribution to FN or MS categories is subjective, but the relevant difference in sensitivity at conventional, CAD and DOUBLE readings observed in the present study for FN and MS cases (CONV=50.2 *vs* 22.3%; CAD=62.6 *vs* 30.7%, DOUBLE=64.8 *vs* 35.7%) confirm the validity of the classification.

The analysis of the accuracy of the CAD system shows a low overall sensitivity as at least one abnormality in one view was marked only in 48.3% of cancer cases. This sensitivity is much less than the 94.1% sensitivity observed with the same algorithm for screen detected cases in a previous study (Ciatto *et al*, 2003). Such a difference may be explained by the different frequency of cancer microcalcifications, for which CAD is more sensitive (Brem *et al*, 2000; Malich *et al*, 2001), in the two series, and by the selection by diagnostic modality: screen detected cases are associated with more easily perceivable abnormalities (that is, why they were detected at screening) as compared to interval cases, particularly those classified as MS at review, which are associated to minor/minimal changes that evidently are less easily perceived also by the CAD algorithm. In fact, the 90.9% sensitivity of CAD for FN cases is similar to that (94.1%) reported for screen detected cases (Ciatto *et al*, 2003), while the 25.0% sensitivity of CAD for MS cases is much lower. Therefore, CAD had a high sensitivity for cases in which it is most likely to assist the radiologist – cancers missed due to oversight errors. It must be also noted that several cases in the present series had only single oblique view. This is likely to have biased CAD sensitivity towards lower values as CAD sensitivity is higher when two instead of one single view is available for analysis.

Similar and expected differences were seen in radiologist sensitivity on a case basis: sensitivity at CONV was significantly higher for FN as compared to MS cases. The detection rate of MS cases was very low, almost in the range of the recall rate of negative cases both at CONV (sensitivity for MS=22.3%, recall rate=16.9%) and at CAD (sensitivity for MS=30.7%, recall rate=23.9%), suggesting that almost the same specificity and detection rate of MS cases would have been achieved if 20–25% of cases in the set would have been recalled at random. As the definition of MS categories implies a very low intrinsic chance of detection, the impact of CAD in improving the detection of interval cancers should be reasonably determined mainly on FN cases. This further stresses the importance of the observed 90.9% sensitivity of CAD for FN cases.

Computer-aided detection system case-based specificity (17.9%) was also low, but the aim of CAD is not diagnosis, rather CAD alerts the reader to specific areas for second review. Computer-aided detection marking is a necessary condition, though not sufficient, to assume that CAD has contributed to increased sensitivity or reduced specificity. While CAD marking alerts the reader, further analysis of the marked area by the reader does not necessarily lead to recall for further assessment. In fact, there were cancers and negative cases marked by CAD but not recalled at CAD reading, as well as lesions not marked by CAD but recalled at CAD reading (sometimes even if not recalled at the previous conventional reading). The latter condition suggests the existence of intraobserver inconsistency, which is expected to affect radiological judgement.

Computer-aided detection proved to be not very sensitive for interval cancers. When the cancer lesion is not marked by CAD, there is the risk that the radiologist may be negatively influenced, and discount previous suspicion arising at conventional reading. This might have occurred in the present study, with 16 cancers not marked by CAD. Out of 304 cancer readings in these cases, suspicion of cancer was reported in 77 cases at CONV, and only in 31 cases at CAD assisted reading. In order to avoid the risk of lesions detected at CONV and not being worked up by the radiologist because CAD did not mark the lesion, CAD was intended to assist radiologists only in detecting ‘additional’ lesions for work-up, not for determining which lesions not to work-up. Thus, we assumed that CAD should only produce additional recalls to assessment, and we considered as CAD-related changes only CAD-marked cases that had been reported as negative at CONV reading and as positive at CAD reading. This gave the maximum expected effect of CAD on sensitivity, which reached statistical significance when compared to CONV, but also resulted in a higher negative effect of CAD on recall rates.

However, the impact of CAD on diagnostic accuracy should not be determined according to the proportion of benign and cancer lesions marked by the system (Warren Burhenne *et al*, 2000), but more appropriately should be based on the effect of CAD on actual recalls (Freer and Ulissey, 2001). Of course, the fact that in the present study CAD marked more than one area per film and more than two areas per case is relevant, as it may imply an extra workload for the reader and possibly influence the reader towards a lower specificity. When reading screening mammograms, and particularly when running a proficiency test, the reader has a tendency to maximise sensitivity accepting an excess of unnecessary recalls. This condition might be further influenced by the high frequency of sites marked for second review by CAD.

The impact of CAD as an adjunct to conventional reading was somehow expected from the evidence of CAD system accuracy: sensitivity was improved for 17 of 19 readers, with an average absolute increase of sensitivity of 9.9%, statistically significant. The absolute increase in sensitivity for FN cases was even higher (12.4%), which makes a 7.0% absolute increase of recall rate fully acceptable. The absolute increase of sensitivity at CAD reading was quite variable among readers, being null for two readers and ranging from +2.8% to +29.1% among the other readers. The individual impact of CAD-assisted reading on sensitivity did not correlate with baseline sensitivity at conventional reading. This finding suggest that the influence of CAD assisted reading on the sensitivity of single radiologists may vary to a major extent and is not easily predictable.

The comparison of CAD and DOUBLE confirms a previous finding on a set of screen-detected cancers (Ciatto *et al*, 2003), suggesting a comparable performance for the two readings. The finding of a slight, insignificant higher sensitivity of DOUBLE and a significant higher specificity of CAD encourages further comparative studies on this aspect. Such studies are urgently needed, considering the difficulties that the recommended practice of routine DOUBLE is facing in Europe, due to high related costs and lack of radiologists. Demonstrating that CAD may be a reliable surrogate to DOUBLE might have a major favourable impact on population-based screening organisation.

In conclusion, in accordance with other reports (Brem *et al*, 2000; Malich *et al*, 2001), the study suggests that CAD has a considerable favourable impact (increased sensitivity) on reading of screening mammograms, which is balanced by a limited negative effect (increased recall rate), and might be currently adopted in the current practice as an adjunct to single reading. In the Florence District programme (Paci *et al*, 1999), the sensitivity of biennial screening (with single reading) of women aged 50–69 years has been calculated on the basis of proportional interval cancer incidence and resulted to be 75%. Based on the expected distribution of interval cancer by error type (FN=11.9%, MS=26.1% and occult=61.9%) (Ciatto *et al*, 1995a, 1995b) and on the results observed in the present study (sensitivity increase at CAD-assisted reading; FN=+12.4%, MS=+8.4%), the absolute increase in screening sensitivity of CAD-assisted reading as compared to single reading might be estimated around +0.9%. Further investigation of CAD by means of controlled longitudinal studies should address cost-effective analysis, which is particularly important in a organised screening scenario. The finding that CAD reading had a similar performance as compared to simulated DOUBLE, particularly to identify FN cases, suggests a possible future use of CAD that needs to be confirmed by prospective studies.

## References

[bib1] Bird RE (1990) Professional quality assurance for mammographic screening programs. Radiology 177: 587–591 10.1148/radiology.177.2.2217807 2217807

[bib2] Bird RE, Wallace TW, Yankaskas BC (1992) Analysis of cancers missed at screening mammography. Radiology 184: 613–617 1509041 10.1148/radiology.184.3.1509041

[bib3] Brem RF, Schoonjans JM, Hoffmeister J, Raza S, Baum JK (2000) Evaluation of breast cancer with a computer-aided detection system by mammographic appearance, histology and lesion size. Radiology 217(P): 400

[bib4] Ciatto S, Ambrogetti D, Catarzi S, Morrone D, Rosselli Del Turco M (1999) Proficiency test for screening mammography: results for 117 volunteer Italian radiologists. J Med Screen 6: 149–151 10572846 10.1136/jms.6.3.149

[bib5] Ciatto S, Rosselli Del Turco M, Ambrogetti D, Catarzi S, Morrone D (1996) Test per la valutazione dell'accuratezza diagnostica nella mammografia. Risultati di 103 test sostenuti da radiologi italiani. Radiol Med 92: 367–371 9045233

[bib6] Ciatto S, Rosselli Del Turco M, Morrone D, Catarzi S, Ambrogetti D, Cariddi A, Zappa M (1995a) Independent double reading of screening mammograms. J Med Screen 2: 99–101 7497164 10.1177/096914139500200209

[bib7] Ciatto S, Rosselli Del Turco M, Risso G, Catarzi S, Bonardi R, Viterbo V, Gnutti P, Guglielmoni B, Pinelli L, Pandiscia A, Navarra F, Lauria A, Palmiero R, Indovina PL (2003) Comparison of standard reading and computer aided detection (CAD) on a national proficiency test of screening mammography. Eur J Radiol 45: 135–138 12536093 10.1016/s0720-048x(02)00011-6

[bib8] Ciatto S, Rosselli Del Turco M, Zappa M (1995b) The detectability of breast cancer by screening mammography. Br J Cancer 71: 337–339 7841050 10.1038/bjc.1995.67 PMC2033595

[bib9] Dijk JAAM, Verbeek ALM, Hendriks JHCL (1993) The current detectability of breast cancer in a mammographic screening program: a review of the previous mammograms of interval and screen-detected cancers. Cancer 72: 1933–1938 8364871 10.1002/1097-0142(19930915)72:6<1933::aid-cncr2820720623>3.0.co;2-n

[bib10] Perry N, Broeders M, de Wolf C, Törnberg S (eds) (2001) European Guidelines for Quality Assurance in Mammographic Screening, 3rd edn, pp 155–158. Luxembourg: European Commission

[bib11] Freer TW, Ulissey MJ (2001) Screening mammography with computer aided detection: prospective study of 12,860 patients in a community breast center. Radiology 220: 781–786 11526282 10.1148/radiol.2203001282

[bib12] Harvey JA, Fajardo LL, Innis CA (1993) Previous mammograms in patients with impalpable breast carcinoma: retrospective vs blinded interpretation. AJR 161: 1167–1172 8249720 10.2214/ajr.161.6.8249720

[bib13] Kirkpatrick A, Thornberg S, Thissen AI (1992) The European Guidelines for Quality Assurance in Mammography Screening, Europe Against Cancer Programme. Brussels: European Community

[bib14] Kopans DB (1998) Breast Imaging, 2nd edn, p 214. Philadelphia: Lippincott-Raven

[bib15] Malich A, Marx C, Facius M, Boehm T, Fleck M, Kaiser WA (2001) Tumor detection rate of a new commercially available computer-aided detection system. Eur Radiol 11: 2454–2459 11734939 10.1007/s003300101079

[bib16] Paci E, Ciatto S, Buiatti E, Cecchini S, Palli D, Rosselli Del Turco M (1999) Early indicators of efficacy of breast screening programmes. Results of the Florence District Programme. Int J Cancer 46: 198–202 10.1002/ijc.2910460209 2384270

[bib17] Thurfjell EL, Lernevall KA, Taube AAS (1994) Benefit of independent double reading in a population-based mammography screening program. Radiology 191: 241–244 8134580 10.1148/radiology.191.1.8134580

[bib18] Warren Burhenne LJ, Wood SA, D'Orsi CJ (2000) Potential contribution of computer aided detection to the sensitivity of screening mammography. Radiology 215: 554–562 10796939 10.1148/radiology.215.2.r00ma15554

